# Hepatic Cerebroside Sulfotransferase Is Induced by PPAR*α* Activation in Mice

**DOI:** 10.1155/2012/174932

**Published:** 2012-05-07

**Authors:** Takefumi Kimura, Takero Nakajima, Yuji Kamijo, Naoki Tanaka, Lixuan Wang, Atsushi Hara, Eiko Sugiyama, Eiji Tanaka, Frank J. Gonzalez, Toshifumi Aoyama

**Affiliations:** ^1^Department of Metabolic Regulation, Institute on Aging and Adaptation, Shinshu University Graduate School of Medicine, 3-1-1 Asahi, Matsumoto 390-8621, Japan; ^2^Department of Gastroenterology, Shinshu University School of Medicine, Matsumoto 390-8621, Japan; ^3^Department of Nephrology, Shinshu University School of Medicine, Matsumoto 390-8621, Japan; ^4^Laboratory of Metabolism, National Cancer Institute, National Institutes of Health, Bethesda, MD 20892, USA; ^5^Department of Nutritional Science, Nagano Prefectural College, Nagano 380-8525, Japan

## Abstract

Sulfatides are one of the major sphingoglycolipids in mammalian serum and are synthesized and secreted mainly from the liver as a component of lipoproteins. Recent studies revealed a protective role for serum sulfatides against arteriosclerosis and hypercoagulation. Although peroxisome proliferator-activated receptor (PPAR) **α** has important functions in hepatic lipoprotein metabolism, its association with sulfatides has not been investigated. In this study, sulfatide levels and the expression of enzymes related to sulfatide metabolism were examined using wild-type (+/+), *Ppara*-heterozygous (+/−), and *Ppara*-null (−/−) mice given a control diet or one containing 0.1% fenofibrate, a clinically used hypolipidemic drug and PPAR**α** activator. Fenofibrate treatment increased serum and hepatic sulfatides in *Ppara* (+/+) and (+/−) mice through a marked induction of hepatic cerebroside sulfotransferase (CST), a key enzyme in sulfatide synthesis, in a PPAR**α**-dependent manner. Furthermore, increases in CST mRNA levels were correlated with mRNA elevations of several known PPAR**α** target genes, and such changes were not observed for other sulfatide-metabolism enzymes in the liver. These results suggest that PPAR**α** activation enhances hepatic sulfatide synthesis via CST induction and implicate CST as a novel PPAR**α** target gene.

## 1. Introduction

Sulfatides are sphingoglycolipids composed of sphingoid, fatty acid, galactose, and sulfate [1] that are distributed in various tissues such as the central nervous system, kidney, liver, and gastrointestinal tract [1–4]. Glycolipids are also present in the serum as one of the major components of lipoproteins [1]. Several studies have revealed a protective role for serum sulfatides against arteriosclerosis and hypercoagulation [5]. Serum levels of sulfatides are markedly decreased in humans with end-stage renal failure [6] but normalize after renal transplantation [7]. However, the precise mechanism regulating serum sulfatide concentrations in humans remains unclear. Previously studies demonstrated that serum sulfatide levels were strongly correlated with hepatic, but not renal, sulfatide levels in mice with protein overload nephropathy, and that decreased serum sulfatide levels were also associated with the downregulation of hepatic expression of cerebroside sulfotransferase (CST), a key enzyme in sulfatide synthesis [8]. These and previous findings suggest the possible participation of hepatic peroxisome proliferator-activated receptor (PPAR) in the regulation of serum and liver sulfatide metabolisms. To examine this possibility, serum and liver sulfatide concentrations and hepatic expression of a series of sulfatide-metabolizing enzymes were analyzed using *Ppara*-homozygous (+/+), *Ppara*-heterozygous (+/−), and *Ppara*-null (−/−) mice fed a control diet or one containing fenofibrate, a typical PPAR*α* activator.

## 2. Materials and Methods

### 2.1. Mice and Treatment

All animal experiments were conducted in accordance with animal study protocols approved by the Shinshu University School of Medicine. Wild-type (+/+), *Ppara *(+/−), and *Ppara* (−/−) mice on a 129/Sv genetic background were generated as described previously [9–11]. These mice were maintained in a pathogen-free environment under controlled conditions (25°C; 12 h light/dark cycle) with tap water *ad libitum *and a standard rodent diet. Twelve-week-old male wild-type (+/+), *Ppara *(+/−), and *Ppara* (−/−) mice weighing 25–30 g were used for the following experiments. Mice of each genotype were randomly divided into two groups (*n* = 6 in each group of the same genotype). One mouse group was treated with a regular diet containing 0.1% fenofibrate (Wako Pure Chemical Industries, Osaka, Japan), and the other group was continued on a regular diet as a control. In an additional experiment, *Ppara *(+/+), *Ppara *(+/−), and *Ppara* (−/−) mice were randomly divided into two groups (*n* = 6 in each group of the same genotype) and were treated with a regular diet with or without 0.5% clofibrate (Wako Pure Chemical Industries). Seven days after commencing treatment, the mice were sacrificed under anesthesia for collection of blood and tissues.

### 2.2. Extraction and Measurement of Lipids

Total lipids in the serum and liver were extracted using the hexane/isopropanol method [12], and serum/liver sulfatides were determined as forms of lysosulfatides (LS; sulfatides without fatty acids) by matrix-assisted laser desorption ionization-time of flight mass spectrometry (MALDI-TOF MS) as previously described [13]. Sulfatides levels were calculated as the sum of the levels of seven LS molecular species: LS-sphingadienine (LS-d18 : 2), LS-(4*E*)-sphingenine (LS-d18 : 1), LS-sphinganine (LS-d18 : 0), LS-4D-hydroxysphinganine (LS-t18 : 0), LS-(4*E*)-icosasphingenine (LS-d20 : 1), LS-icosasphinganine (LS-d20 : 0), and LS-4D-hydroxyicosasphinganine (LS-t20 : 0). Triglyceride (TG) levels in the serum and liver were measured using a Triglyceride *E*-test kit (Wako Pure Chemical Industries).

### 2.3. Immunoblot Analysis

Liver nuclear and cytosolic fractions were prepared from each mouse using NE-PER Nuclear and Cytoplasmic Extraction Regents (Thermo Fisher Scientific, Rockford, IL, USA) [14], and their protein concentrations were determined with a BCA protein assay kit (Thermo Fisher Scientific) [15]. Nuclear fractions (10 *μ*g protein) were used for immunoblot analysis of PPARs and TATA box-binding protein (TBP). For detection of other proteins, cytosolic fractions (5 *μ*g protein) were employed. Proteins were separated using sodium dodecyl sulfate-polyacrylamide gel electrophoresis and transferred to nitrocellulose membranes. After blocking, the membranes were incubated with primary antibodies followed by alkaline phosphatase-conjugated secondary antibodies [16–18]. Primary antibodies against long-chain acyl-CoA synthase (LACS), liver fatty acid-binding protein (L-FABP), and medium-chain acyl-CoA dehydrogenase (MCAD) were prepared as described previously [19–21]. Antibodies against other proteins were purchased commercially: cerebroside sulfotransferase (CST) from Abnova (Taipei, Taiwan), arylsulfatase A (ARSA) from Everest Biotech (Oxfordshire, UK), TBP from Abcam (Cambridge, UK), and ceramide galactosyltransferase (CGT), galactosylceramidase (GALC), microsomal transfer protein (MTP), PPAR*α*, PPAR*β*/*δ*, PPAR*γ*, and actin from Santa Cruz Biotechnology (Santa Cruz, CA, USA). TBP and actin were used as loading controls for nuclear and cytosolic protein extracts, respectively. Band intensities were measured densitometrically, normalized to those of TBP or actin, and then expressed as fold changes relative to those of *Ppara* (+/+) mice treated with a control diet.

### 2.4. Analysis of mRNA

Total liver RNA was extracted using an RNeasy Mini Kit (QIAGEN, Hilden, Germany), and samples of 2 *μ*g of RNA were reverse-transcribed using oligo-dT primers and SuperScript II reverse transcriptase (Invitrogen Corporation, Carlsbad, CA, USA). Levels of mRNA were quantified by real-time polymerase chain reaction (PCR) using an SYBR Premix Ex Taq II (Takara Bio, Otsu, Japan) on a Thermal Cycler Dice TP800 system (Takara Bio) [10, 16]. Specific primers were designed by Primer Express software (Applied Biosystems, Foster City, CA, USA) as shown in Table 1. The mRNA levels of glyceraldehyde-3-phosphate dehydrogenase (GAPDH) were used as an internal control. Measurements of mRNA levels were normalized to those of GAPDH and then expressed as fold changes relative to those of *Ppara* (+/+) mice treated with a control diet.

### 2.5. Assays for DNA-Binding Activity of PPARs

The DNA-binding activity of nuclear PPAR*α*PPAR*β*/*δ*, and PPAR*γ* was determined using PPAR*α*, PPAR*β*/*δ*, and PPAR*γ* Transcription Factor Assay kits (Cayman Chemical, Ann Arbor, MI, USA) [22–24], respectively. These assays are based on an enzyme-linked immunosorbent assay using PPAR response element (PPRE) immobilized microplates and specific PPAR antibodies, thus offering similar results to those from the conventional radioactive electrophoretic mobility shift assay. DNA-binding assays were carried out according to the manufacturer's instructions using nuclear extracts (50 *μ*g protein) prepared as described previously. Results are expressed as fold changes relative to those of *Ppara* (+/+) mice treated with a control diet.

### 2.6. Statistical Analysis

All data are expressed as mean ± standard deviation (SD). Statistical analysis was performed using one-way ANOVA with Bonferroni correction (SPSS Statistics 17.0; SPSS Inc, Chicago, IL, USA). Correlation coefficients were calculated using Spearman's rank correlation analysis. A probability value of less than 0.05 was considered to be statistically significant.

## 3. Results

### 3.1. Fenofibrate Increased Serum/Liver Sulfatides in a PPAR*α*-Dependent Manner

**Figure 1:**
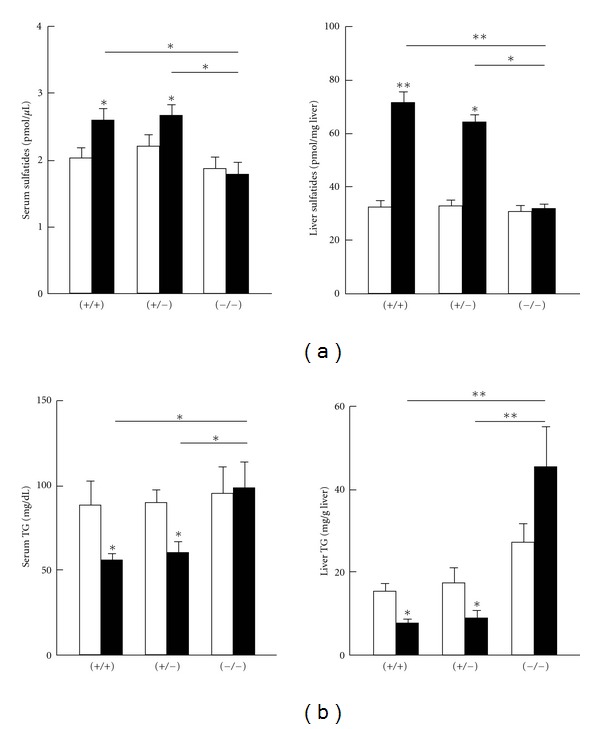
Changes in serum and hepatic levels of sulfatides (a) and TG (b). *Ppara* (+/+), (+/−), and (−/−) mice were treated without (open bars) or with (closed bars) 0.1% fenofibrate for 7 days. Results are expressed as mean ± SD (*n* = 6/group). *P < 0.05; **P < 0.01.

Fenofibrate treatment increased serum, and more notably liver, sulfatide concentrations in *Ppara* (+/+) and (+/−) mice only (Figure 1(a)). However, the increases in serum/liver sulfatides were not detected in *Ppara* (−/−) mice with fenofibrate treatment. These results demonstrate that fenofibrate increases serum/liver sulfatide levels in a PPAR*α*-dependent manner. The treatment did not affect the composition of sulfatides (Table 2). Fenofibrate also decreased serum/liver TG levels in a PPAR*α*-dependent manner (Figure 1(b)), which was in agreement with previous reports [25, 26].

### 3.2. Fenofibrate Upregulated Hepatic CST in a PPAR*α*-Dependent Manner

**Figure 2:**
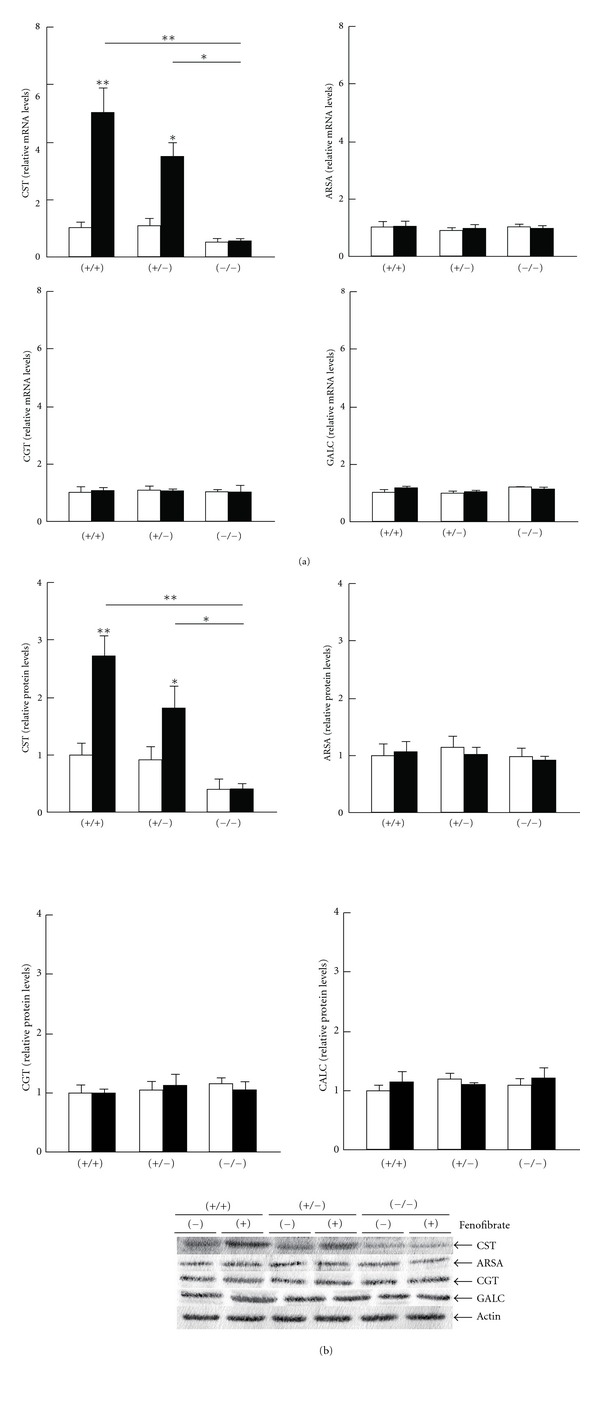
Changes in hepatic expression of sulfatide-metabolizing enzymes by fenofibrate treatment. Open and closed bars indicate mice treated without or with 0.1% fenofibrate, respectively. Data are expressed as mean ± SD (*n* = 6/group). *P < 0.05; **P < 0.01. (a) The mRNA levels of CST, ARSA, CGT, and GALC. Hepatic mRNA levels were normalized to those of GAPDH and then expressed as fold changes relative to those of *Ppara* (+/+) mice treated with a control diet. (b) Immunoblot analysis. Actin was used as the loading control. Band intensities were measured densitometrically, normalized to those of actin, and then expressed as fold changes relative to those of *Ppara* (+/+) mice treated with a control diet.

We assessed several major hepatic sulfatide-metabolizing enzymes to determine the mechanistic basis of the changes observed in sulfatide concentrations. CST and ARSA, respectively, catalyze the forward and reverse reactions from galactosylceramides to sulfatides, and a similar relationship exists for CGT and GALC in the synthesis of galactosylceramides from ceramides [8]. Fenofibrate treatment significantly increased levels of mRNA encoding CST in *Ppara* (+/+) and (+/−) mice (Figure 2(a)), with the extent of induction higher in the *Ppara* (+/+) group. Upregulation of CST expression by fenofibrate was not observed in *Ppara* (−/−) mice. PPAR*α*-dependent increases in CST mRNA corresponded to increases in CST protein levels (Figure 2(b)). Fenofibrate treatment did not affect expression of the other sulfatide-metabolizing enzymes, ARSA, CGT, and GALC, at either the mRNA or the protein level (Figure 2). Since hepatic CST mRNA levels were strongly correlated with sulfatide levels in the serum (*r* = 0.886, *P* = 0.019) and liver (*r* = 0.943, *P* = 0.005), the increased serum/liver sulfatide levels found after treatment were viewed as mainly due to the significant induction of hepatic CST.

### 3.3. Hepatic CST Was Induced by PPAR*α* Activation

**Figure 3:**
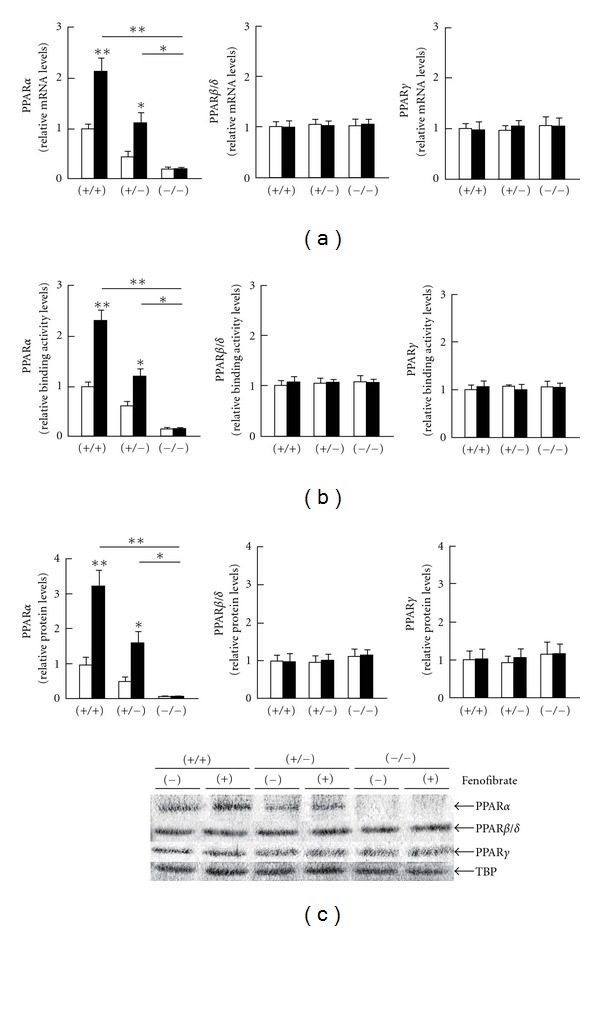
Changes in hepatic expression of PPARs by fenofibrate treatment. Open and closed bars indicate mice treated without or with 0.1% fenofibrate, respectively. Data are expressed as mean ± SD (*n* = 6/group). *P < 0.05; **P < 0.01. (a) The mRNA levels of PPARs. Hepatic mRNA levels were normalized to those of GAPDH and then expressed as fold changes relative to those of *Ppara* (+/+) mice treated with a control diet. (b) PPAR-binding activity based on an enzyme-linked immunosorbent assay. Detailed protocols are described in Section 2. Results are expressed as fold changes relative to those of *Ppara* (+/+) mice treated with a control diet. (c) Immunoblot analysis. TBP was used as the loading control. Band intensities were measured densitometrically, normalized to those of TBP, and then expressed as fold changes relative to those of *Ppara* (+/+) mice treated with a control diet.

**Figure 4:**
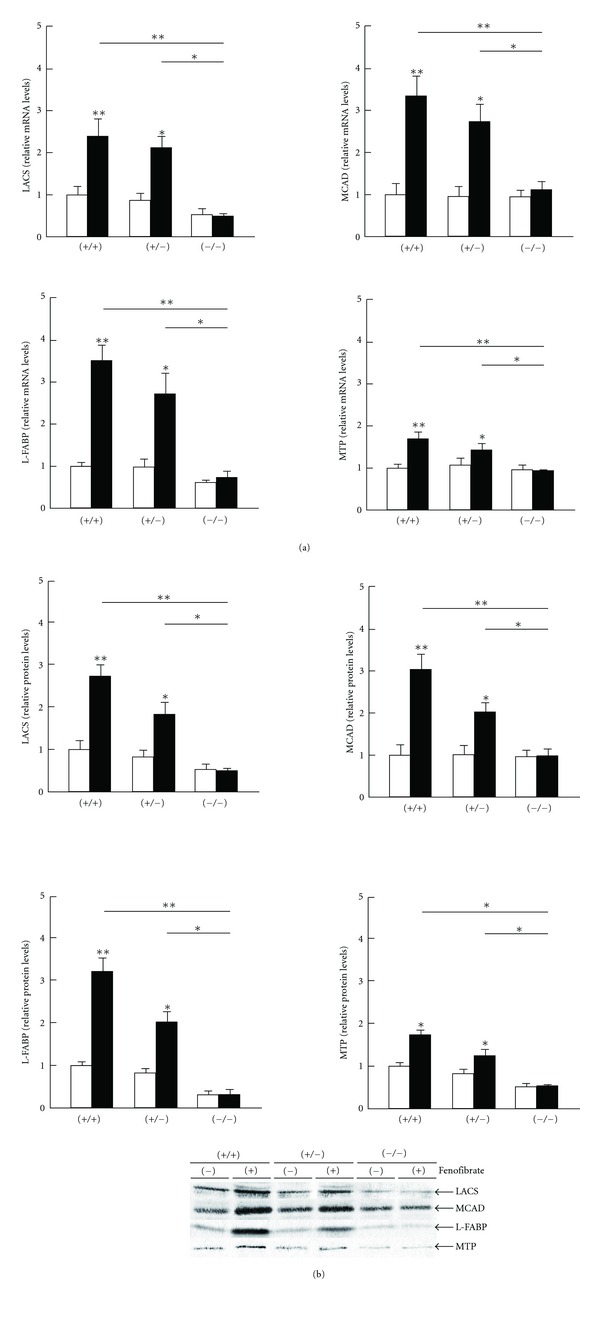
Changes in hepatic expression of conventional PPAR*α* target genes by fenofibrate treatment. Open and closed bars indicate mice treated without or with fenofibrate, respectively. Data are expressed as mean ± SD (*n* = 6/group). *P < 0.05; **P < 0.01. (a) Analysis of mRNA. Hepatic mRNA levels were normalized to those of GAPDH and then expressed as fold changes relative to levels of *Ppara* (+/+) mice treated with a control diet. (b) Immunoblot analysis. Band intensities were measured densitometrically, normalized to those of actin, and then expressed as fold changes relative to those of *Ppara* (+/+) mice treated with a control diet.

**Figure 5:**
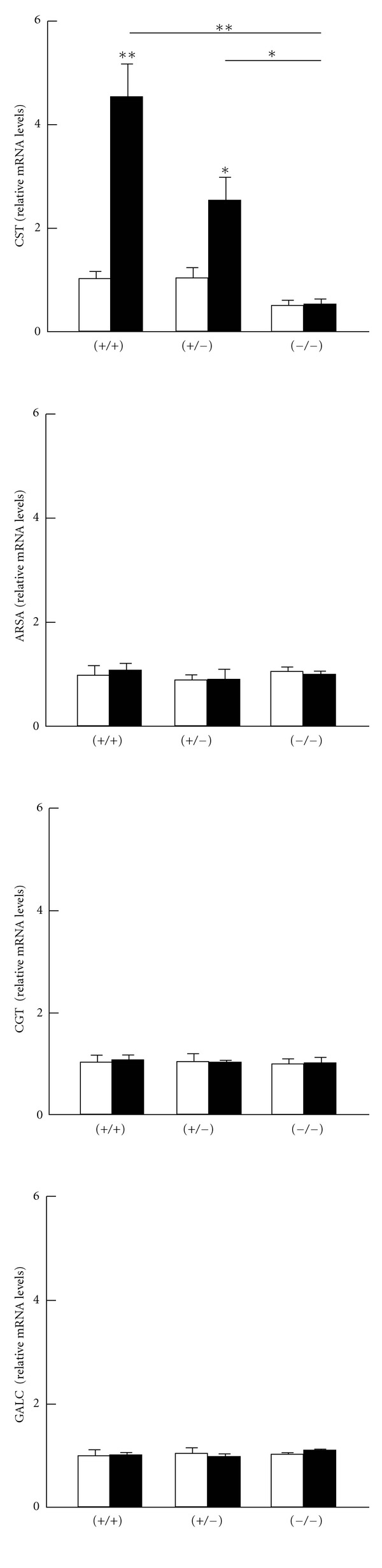
PPAR*α*-dependent induction of CST mRNA levels by clofibrate treatment. Open and closed bars indicate mice treated without or with 0.5% clofibrate, respectively. Data are expressed as mean ± SD (*n* = 6/group). *P < 0.05; **P < 0.01.

 As expected, fenofibrate treatment significantly enhanced hepatic expression of PPAR*α* and several representative PPAR*α* target genes, including LACS, MCAD, L-FABP, and MTP (Figures 3 and 4) [27–29]. The DNA binding activity levels of PPAR*α* were also elevated by fenofibrate (Figure 3(b)). The treatment did not influence the expression and activity of PPAR*β*/*δ* or PPAR*γ* (Figure 3), nor did it affect levels of CST mRNA or protein in the livers of *Ppara* (−/−) mice (Figure 2). The mRNA levels of CST were strongly correlated with those of PPAR*α* target gene products (*r* = 0.886, *P* = 0.019 for LACS; *r* = 0.928, *P* = 0.008 for MCAD; *r* = 0.943, *P* = 0.005 for L-FABP; and *r* = 0.943, *P* = 0.005 for MTP). PPAR*α*-dependent induction of CST mRNA levels was also observed in mice treated with clofibrate, another typical PPAR*α* activator (Figure 5). These results indicated that the induction of hepatic CST was closely associated with PPAR*α* activation in mice.

## 4. Discussion

The present study revealed that fenofibrate treatment increased serum/liver sulfatide levels and the expression of hepatic CST mRNA and protein through PPAR*α* activation. As CST mRNA levels were closely correlated with those of four known PPAR*α* target genes, these findings suggest that CST may be a novel PPAR*α* target gene candidate.

While CST is a key enzyme in sulfatide metabolism, little is known about its transcriptional regulation. We recently reported that an increase in hepatic oxidative stress downregulated CST expression in mice [8], although the mechanism remains unclear. A search for putative PPRE regions in the mouse CST gene [30, 31] revealed several candidates: −1,434/−1,422 (AGGTCTAAGGGCA), −1,202/−1,190 (TGGACTTTGCCCT), and −896/−884 (AGGACAAAGAGCA) from exon 1a; −1,499/−1,487 (AGGCTACAGTTCA) from exon 1e; and −1,569/−1,557 (AGGTCAGAGCACA) and −302/−290 (AGGACAGAGCCCA) from exon 1f. These regions may be useful for analysis in future in vitro experiments.

 The degree of increases in serum sulfatides was lower than that in hepatic sulfatides by fenofibrate treatment (1.27-fold in the serum versus 2.20-fold in liver in* Ppara* (+/+) mice and 1.22-fold in the serum versus 1.95-fold in the liver of *Ppara* (+/−) mice). Sulfatides synthesized in the liver are secreted into the blood together with TG as a component of very-low-density lipoprotein (VLDL) [32]. Thus, hepatic TG content was reduced by fenofibrate treatment probably due to the enhanced of mitochondrial *β*-oxidation ability resulting in a reduction of hepatic VLDL synthesis as seen in other experiments using cultured hepatocytes [33]. Further studies are required to determine sulfatide metabolism in the serum and liver since they are significantly influenced by numerous pathophysiological events and treatments, including acute kidney injury [8, 34], clofibrate pretreatment [35], chronic kidney disease [6], and kidney transplantation [7].

The role of PPAR*α* has been clarified in several liver diseases. For instance, PPAR*α* is downregulated in alcoholic liver disease [11, 36] as well as after liver transplantation[37]. Persistent activation of PPAR*α* ameliorates hepatic steatosis and inflammation in mice but may also induce hepatocarcinogenesis [10]. The association between liver disease and sulfatide metabolism may be of interest for further research.

Lastly, several animal studies have uncovered a protective role for serum sulfatides against arteriosclerosis and hypercoagulation [5]. We also reported a close relationship between lower serum sulfatide concentrations and higher incidences of cardiovascular disease in patients with end-stage renal failure [6], in whom sulfatide levels returned to normal following kidney transplantation [7]. Accordingly, increasing or maintaining serum sulfatide levels using fibrates may be useful in reducing the risk of cardiovascular events, which is consistent with the known beneficial effect of fibrates seen in randomized controlled studies [38]. Furthermore, these findings show a need to examine sulfatide metabolism in cardiomyocytes, endothelial cells, and vascular smooth cells to disclose any novel protective roles of PPAR*α* in cardiovascular inflammation and atherosclerosis, particularly in relation to CST upregulation.

## Figures and Tables

**Table 1:** Primer pairs used for the RT-PCR.

Gene	GeneBank accession number		Primer sequence
ARSA	NM_009713	F	5′-ACCACCCCTAACCTGGATCAGT-3′
		R	5′-ATGGCGTGCACAGAGACACA-3′
CGT	NM_011674	F	5′-TGGGTCCAGCCTATGGATGT-3′
		R	5′-GCAGCGTTGGTCTTGGAAAC-3′
CST	NM_016922	F	5′-ATGGCCTTCACGACCTCAGA-3′
		R	5′-CGGTCTTGTGCGTCTTCATG-3′
GALC	NM_008079	F	5′-GAGTGAGAATCATAGCGAGCGATA-3′
		R	5′-AGTTCCTGGTCCAGCAGCAA-3′
GAPDH	M32599	F	5′-TGCACCACCAACTGCTTAG-3′
		R	5′-GGATGCAGGGATGATGTTCTG-3′
LACS	NM_007981	F	5′-TCCTACGGCAGTGATCTGGTG-3′
		R	5′-GGTTGCCTGTAGTTCCACTTGTG-3′
L-FABP	NM_017399	F	5′-GCAGAGCCAGGAGAACTTTGAG-3′
		R	5′-TTTGATTTTCTTCCCTTCATGCA-3′
MCAD	NM_007382	F	5′-TGCTTTTGATAGAACCAGACCTACAGT-3′
		R	5′-CTTGGTGCTCCACTAGCAGCTT-3′
MTP	NM_008642	F	5′-GAGCGGTCTGGATTTACAACG-3′
		R	5′-GTAGGTAGTGACAGATGTGGCTTTTG-3′
PPAR*α*	NM_011144	F	5′-CCTCAGGGTACCACTACGGAGT-3′
		R	5′-GCCGAATAGTTCGCCGAA-3′
PPAR*β*/*δ*	XM_128500	F	5′-TCAACATGGAATGTCGGGTT-3′
		R	5′-ATACTCGAGCTTCATGCGGATT-3′
PPAR*γ*	NM_011146	F	5-TTCCACTATGGAGTTCATGCTTGT-3′
		R	5′-TCCGGCAGTTAAGATCACACCTA-3′

F: forward sequence; R: reverse sequence.

**Table 2:** Composition of serum and liver sulfatides.

	Serum						Liver					
	(+/+)		(+/−)		(−/−)		(+/+)		(+/−)		(−/−)	
	(−)	(+)	(−)	(+)	(−)	(+)	(−)	(+)	(−)	(+)	(−)	(+)
d18 : 2	7	9	8	7	8	7	12	11	12	13	11	12
d18 : 1	34	31	33	36	33	35	29	30	30	28	30	31
d18 : 0	11	11	12	10	11	10	11	10	12	11	10	12
t18 : 0	7	9	8	7	8	7	6	6	6	5	7	6
d20 : 1	8	11	9	8	9	8	12	11	10	12	10	10
d20 : 0	5	7	6	6	6	6	10	9	9	10	9	8
t20 : 0	28	22	24	26	25	27	20	23	21	21	23	21

(−): mice treated with a control diet; (+): mice treated with fenofibrate; d18 : 2: sphingadienine; d18 : 1: (4*E*)-sphingenine; d18 : 0: sphinganine; t18 : 0: 4D-hydroxysphinganine; d20 : 1: (4*E*)-icosasphingenine; d20 : 0: icosasphinganine; t20 : 0: 4D-hydroxyicosasphinganine.

Data are expressed as percentages.

## Abbreviations

ARSA: Arylsulfatase A
CGT: Ceramide galactosyltransferase
CST: Cerebroside sulfotransferase
GALC: Galactosylceramidase
GAPDH: Glyceraldehyde-3-phosphate dehydrogenase
LACS: Long-chain acyl-CoA synthase
L-FABP: Liver fatty acid-binding protein
LS: Lysosulfatides
MCAD: Medium-chain acyl-CoA dehydrogenase
MTP: Microsomal triglyceride transfer protein
PCR: Polymerase chain reaction
PPAR: Peroxisome proliferator-activated receptor
PPRE: PPAR response element
TG: Triglycerides.
